# The Role of Self-Efficacy and Activity Patterns in the Physical Activity Levels of Women with Fibromyalgia

**DOI:** 10.3390/biology12010085

**Published:** 2023-01-05

**Authors:** Ana Myriam Lavín-Pérez, Daniel Collado-Mateo, Alexander Gil Arias, Lorena Gutiérrez, Carmen Écija, Patricia Catalá, Cecilia Peñacoba

**Affiliations:** 1 Centre for Sport Studies, Rey Juan Carlos University, Fuenlabrada, 28943 Madrid, Spain; 2 GO fitLAB, Ingesport, 28003 Madrid, Spain; 3 Department of Psychology, Rey Juan Carlos University, 28922 Alcorcón, Spain

**Keywords:** self-efficacy, accelerometry, sedentarism, chronic pain

## Abstract

**Simple Summary:**

Self-efficacy has been identified as a crucial variable to reduce sedentarism in women with fibromyalgia. The present study aimed to evaluate the role of self-efficacy, the impact of fibromyalgia, and activity patterns on the objective physical activity levels. One hundred and twenty-three women with fibromyalgia participated in this cross-sectional study. Physical activity levels were assessed with accelerometers, while self-efficacy, activity patterns, and fibromyalgia impact were evaluated through questionnaires. Results revealed that self-efficacy for physical activity was directly related to light, moderate, and vigorous physical activity, as well as inversely related to sedentary time. Self-efficacy for walking and light physical activity seems to be more relevant than self-efficacy for moderate and vigorous physical activity to achieve higher levels of physical activity.

**Abstract:**

Keeping high levels of physical activity is a challenge among chronic patients. In this regard, self-efficacy has been identified as a crucial variable to reduce sedentarism and physical inactivity in women with fibromyalgia. The current study aimed to evaluate the associations among objective physical activity levels, self-efficacy, activity patterns, and the impact of the disease, as well as to compare those variables between women with fibromyalgia with different self-efficacy levels. For this purpose, in this cross-sectional study, the physical activity levels of 123 women with fibromyalgia were assessed by accelerometers, together with self-efficacy, the impact of the disease, and activity patterns. Results revealed that self-efficacy for light or moderate physical activity was directly related to light (*p* < 0.01), moderate (*p* < 0.01), and vigorous physical activity (*p* < 0.05), as well as inversely related to sedentary time (*p* < 0.01). Moreover, the main differences were observed between those with low self-efficacy levels and the rest of the sample, while there were no differences between the high and the medium self-efficacy groups (*p* > 0.05). Thus, self-efficacy for walking and light physical activity seems to be more relevant than self-efficacy for moderate and vigorous physical activity to achieve higher levels of physical activity.

## 1. Introduction

Fibromyalgia is a chronic musculoskeletal disorder that involves widespread and persistent pain and is associated with many different physical and psychological symptoms, such as fatigue, sleep disturbance, cognitive problems, anxiety, or depression [1]. Physical inactivity has a negative impact on the quality of life and the activities of daily living of people with fibromyalgia [2]. In fact, women with fibromyalgia often show reduced physical function due to low physical activity levels in their leisure time and sedentarism [3,4].

The treatment for people with fibromyalgia is a challenge for health professionals and, to date, the most recommended intervention is a combination of different approaches, including pharmacological and non-pharmacological treatments, but these last ones may be more effective than drugs [5,6]. Among non-pharmacological interventions, physical exercise has been suggested to be the most effective [6]. Psychological therapies, especially cognitive-behavioral therapy, can also lead to benefits for pain, mood, disability, or coping, among others [7,8]. 

Self-efficacy is often low in women with fibromyalgia [9]. It can be defined as a construct of one’s belief in obtaining control over behavior and environment to achieve a goal [10], and it is a crucial variable for women with this disease since it is related to the achievement of physical activity goals [11]. Along with perceived barriers and intention to exercise, self-efficacy is the main predictor for increasing physical activity in patients with fibromyalgia [12]. It is also related to the perception of their symptoms, since, apart from the objective physical problems associated with fibromyalgia, the perception of these patients on their own physical condition is even worse than the actual performance in physical tasks, which has been associated with catastrophism and self-efficacy [13].

Pain is the main symptom of fibromyalgia and strongly affects the achievement of any goal that patients can aim for [14], including, for example, their professional involvement [15]. Furthermore, pain levels have been associated with moderate to vigorous and overall physical activity [16,17], as well as physical function [18]. Thus, people with fibromyalgia must deal with the incompatibility of different goals and their pain and must choose between achievement and pain avoidance goals [19,20,21]. When the prioritization is avoidance, there is an increased risk of disability and increased severity of the symptoms [22,23].

There has been a large increase in the number of publications focusing on fibromyalgia and physical activity or physical exercise. In fact, in the Web of Science (a database that includes Current Contents Connect, Derwent Innovations Index, Korean Journal Database, Medline, Russian Science Citation Index, and SciELO Citation Index), more than three out of four studies on this topic have been published from 2010 to date. The association between physical activity levels, self-efficacy, and activity avoidance in women with fibromyalgia has been previously examined [24] and two different models were suggested according to the intensity of the physical activity. The first one, for low physical activity, involved the physical activity goal preference and the activity avoidance behavior as a mediator of self-efficacy to explain physical activity levels, whereas for moderate to vigorous physical activity, self-efficacy itself was the only predictor. In this regard, the current study was designed to provide further information about the relationship among these variables by adding the sedentary time, other activity patterns (pain avoidance, task-contingent persistence, excessive persistence, pain-contingent persistence, pacing to increase activity levels, pacing to conserve energy for valued activities, and pacing to reduce pain), and making comparisons according to the overall self-efficacy levels, despite several barriers, and for different types of physical activity.

Therefore, the current study aimed to evaluate the associations among objective physical activity levels, self-efficacy, activity patterns, and the impact of the disease, as well as to compare those variables between women with fibromyalgia with different self-efficacy levels. This study hypothesized that self-efficacy and activity patterns would be related to the physical activity levels of women with fibromyalgia. Furthermore, it was expected that those patients with high self-efficacy levels for all types of physical activity would be more active than those with high levels for only walking and light physical activity, while these patients would also be more active than those with low self-efficacy for all types of physical activity.

## 2. Materials and Methods

### 2.1. Participants

A total of 123 women with fibromyalgia were involved in the current study. All of them had been diagnosed according to the criteria of the American Rheumatology Association [25]. Table 1 shows that the mean age of the sample size was 56.16 (SD = 9.07). Regarding the body mass index (BMI), the mean value of the participants was above the recommended ideal BMI, being >25 but <30. The mean years suffering from pain and fatigue problems were 27.09 (SD = 13.45) and 22.44 (SD = 14.50), respectively. Furthermore, the impact of the disease, assessed using the Spanish version of the revised Fibromyalgia Impact Questionnaire [26], was 68.70 (SD = 18.73).

### 2.2. Design and Procedure

The current study followed the ethical guidelines of the Declaration of Helsinki and was reviewed and approved by the Bioethics Committee of Rey Juan Carlos University (Reference PI17/00858). In this sense, an anonymous code was assigned to each participant to maintain. Accordingly, all women signed the informed consent before being included in the study.

Recruited participants belonged to different fibromyalgia associations in the communities of Madrid and Castilla-La Mancha (Spain). The inclusion criteria were women with fibromyalgia older than 18 years, who had read and signed the informed consent, had the medical recommendation of walking, and did not present physical impairments that could limit their physical activity levels (i.e., musculoskeletal injuries such as sprains, ligament tears, bone fractures, etc.).

In this cross-sectional prospective design, accelerometer variables were recorded for seven consecutive days, starting just after the evaluation of the other variables was completed.

### 2.3. Variables and Instruments

Participants answered socio-demographic questions and completed the revised Fibromyalgia Impact Questionnaire (FIQR) in the Spanish version [26], the Self-efficacy for Physical Activity Scale (SEPAS) [27], and the Activity Pattern Scale [28]. They also wore an ActiGraph (Pensacola, FL, USA) GT3X-BT accelerometer to evaluate their physical activity levels.

#### 2.3.1. Fibromyalgia Impact

The FIQR [29] was used to evaluate the impact of the disease on patients. This tool is an updated version of the original Fibromyalgia Impact Questionnaire [30,31], and the Spanish version was developed by Salgueiro et al [26]. It is comprised of three domains: physical function (score from 0 to 30), the overall impact of fibromyalgia (score from 0 to 20), and symptoms domain (which includes 10 different symptoms and the score ranges from 0 to 50). It is a reliable, valid, and widely used questionnaire in Spanish women with fibromyalgia, with an intraclass correlation coefficient (ICC) higher than 0.70 [26].

#### 2.3.2. Self-Efficacy for Physical Activity

The SEPAS was adapted by López-Roig et al. [27] from the original version by Fernández Cabrera et al [32]. This questionnaire assessed the confidence of the ability to perform different types of physical activity despite a number of barriers. It is composed of 35 items focused on several activities, including (a) walking while taking advantage of doing other activities (5 items); (b) doing physical activity at three different intensities: light (5 items), moderate (5 items), and vigorous (5 items); and (c) walking vigorously for 30 (5 items), 60 (5 items), or 90 min (5 items). The five items for each type of physical activity are related to five common barriers, which are the most frequently reported by women with fibromyalgia: pain, fatigue, bad weather, feeling stressed, sad, and worried, and having a bad day due to fibromyalgia. Higher scores mean higher self-efficacy. The internal consistency of the total scale was α = 0.96 [32].

#### 2.3.3. Activity Patterns

The Activity Pattern Scale was validated by Esteve et al. [28] to be used in chronic pain patients, and by López-Roig et al. [33] in women with fibromyalgia. This valid and reliable tool is composed of 24 items grouped into 8 factors: pain avoidance (α = 0.80), activity avoidance (α = 0.73), task-contingent persistence (α = 0.83), excessive persistence (α = 0.73), pain-contingent persistence (α = 0.82), pacing to increase activity (α = 0.76), pacing to conserve energy (α = 0.83), and pacing to reduce pain (α = 0.76) [28]. Answers have a range between 0 (not at all) and 4 (always).

#### 2.3.4. Physical Activity

Participants’ objective physical activity (PA) was recorded with an ActiGraph (Pensacola, USA) GT3X-BT accelerometer. The PA was measured by placing the accelerometer on the wrist of each participant for 7 consecutive days, starting on the same day that they received the accelerometer. Participants were instructed to always wear the accelerometer, except in water activities, such as, for instance, having a shower or swimming. Activity counts were measured at 100 Hz and stored at an epoch length of 15 s [34]. Participants who wore the accelerometer for less than 5 days were excluded from the analysis. The accelerometer was positioned around the hip, under clothing, and fastened with an elastic belt. If any problem or question was detected during its use, participants could contact the investigation team. Data was downloaded directly from each accelerometer and the sign cleaning and analysis were assessed using a tri-axial, GENEActiv PC Software [35]. All wearing days were considered in the analysis. The data was extracted using GENEActiv software and then collapsed using the following equation:

(∑ |√𝑥2 + 𝑦2 + 𝑧2 − 𝑔|)(1)

The type of PA performed was analyzed regarding the sum of vector magnitudes according to the following cut-points: <191.8 epochs was considered sedentary behavior, between 191.8 and 281.5 epochs participants were developing light PA, epochs from 281.5 to 595 were classified as moderate PA, and vigorous PA was carried out when epochs were higher than 595 (Dillon et al., 2016). Subsequently, by dividing the epochs obtained between 4, we got the minutes dedicated to each of the PA modalities.

### 2.4. Statistical Analysis

The database was analyzed using the Statistical Package for the Social Sciences (SPSS) version 23 (IBM, Armonk, NY, USA). Normality was checked through the Kolmogorov- Smirnov and the Shapiro-Wilk tests. Most variables did not follow a normal distribution, so the non-parametric approach was chosen.

The Spearman’s rank correlation coefficient test was used to evaluate the association between physical activity data from the accelerometers and the remaining variables: impact of fibromyalgia, self-efficacy, and activity patterns.

Due to the well-known relevance of self-efficacy, a k-means cluster analysis was conducted. Three groups were created by introducing four variables from the SEPAS: self-efficacy for walking while taking advantage of doing other activities, self-efficacy for light physical activity, for moderate physical activity, and for vigorous physical activity. Differences between the three cluster groups were checked through the Kruskal Wallis one-way analysis of variance, while pairwise comparisons were performed through Bonferroni.

## 3. Results

Correlations showed that the time elapsed from the diagnosis of the disease was significantly and directly associated with sedentary time (*p* = 0.009) and inversely related to moderate physical activity (*p* = 0.001) (see Table 2). Sedentary time was also directly related to the overall impact (*p* = 0.002) and the functionality (*p* < 0.001) assessed using the FIQR, as well as the total score of the questionnaire (*p* = 0.003). Moreover, the different types of physical activity were inversely related to the overall impact of the disease and the functionality measured by FIQR, and the total score of FIQR (*p* < 0.05).

Self-efficacy for walking while taking advantage of doing other activities, and for physical activity and light, moderate, and vigorous were significantly and directly related to light (*p* < 0.05), moderate (*p* < 0.01), and vigorous (*p* < 0.05) physical activity assessed using accelerometers, as well as inversely related to the sedentary time (*p* < 0.01) (See Table 3).

Regarding the activity avoidance patterns (Table 3), the mean avoidance was directly associated with sedentary time (*p* = 0.001) and inversely associated with light (*p* = 0.006), moderate (*p* = 0.001), and vigorous physical activity (*p* = 0.026). Similarly, the mean persistence was inversely related to sedentary time (*p* = 0.002) and directly associated with light (*p* = 0.001) and moderate physical activity (*p* = 0.036). The mean pacing was not significantly related to any type of physical activity (*p* > 0.05), while pacing pain reduction was only significantly associated with light physical activity (*p* = *0*.007) (inversely) and sedentary time (*p* = 0.032) (directly). 

A cluster analysis was conducted to compare patients according to their self-efficacy. Three groups were created, as shown in Table 4. Based on the descriptive values of the three groups, the grouping involved one group with values higher than 5 for all types of activity (high self-efficacy group), another group with high values for light physical activity and walking, but low for moderate and vigorous physical activity (medium self-efficacy group), and the third group with low values for all types of physical activities (low self-efficacy group).

Differences among the three groups from the cluster analysis reported significant differences in all variables extracted from the accelerometers (*p* < 0.05) (See Table 5). Exploring the differences in those variables among the three subgroups, there were no significant differences between the high self-efficacy group (i.e., the group with high self-efficacy for walking and light, moderate, and vigorous physical activity) and the medium self-efficacy group (i.e., the group with high self-efficacy for walking and light physical activity, but low for moderate and vigorous physical activity) (*p* > 0.05). However, the low self-efficacy group (the group with low scores for the four variables) reported lower physical activity levels (*p* < 0.05).

Regarding the activity patterns, there were significant differences among groups in the mean avoidance (*p* = 0.002) and the mean persistence (*p* = 0.28), with the poorest score achieved by the group with lower self-efficacy. On the other hand, no significant differences were observed for the mean pacing (*p* = 0.356). As happened with the physical activity levels, there were no significant differences between the high self-efficacy group and the medium self-efficacy group (*p* > 0.05).

Similarly, regarding the impact of fibromyalgia, there were significant differences between those in the group with low self-efficacy and the other two groups (*p* < 0.05), but there were no significant differences between the high and the medium self-efficacy groups (*p* > 0.05).

## 4. Discussion

The current study aimed to explore the relationship between self-efficacy and objective physical activity, assessed using accelerometers in women suffering from fibromyalgia. The role of the activity patterns and the impact of the disease were also analyzed. The main results showed that self-efficacy for walking while taking advantage of doing other activities and for physical activity (light, moderate, and vigorous) were significantly and directly related to light, moderate, and vigorous physical activity, as well as inversely related to sedentary time. This is in line with the main hypothesis of the current study. 

Furthermore, the main differences were observed between those with low self-efficacy levels and the rest of the sample, while there were no differences between the high and the medium self-efficacy groups. This was not expected since we hypothesized that there would be differences between the low and the medium self-efficacy groups, but also between the medium and the high self-efficacy groups. However, this finding can be explained since these last two groups differed by less than 10% in the self-efficacy for walking and light physical activity, with larger differences in the self-efficacy for moderate and vigorous physical activity. Given that the number of minutes of light physical activity was almost twice the combined minutes for moderate and vigorous physical activity, it seems plausible that differences in the self-efficacy for light physical activity are more relevant than differences in moderate or vigorous physical activity. Therefore, an intervention to increase self-efficacy should be recommended for those patients with low baseline values in self-efficacy for light physical activity. These results are in line with previous studies that showed the relevance of self-efficacy to explain the large gap between the intention to do physical activity and the actual performance [9,36], or the reduction in disability [37].

Regarding avoidance behaviors, the current study found that activity avoidance and pain avoidance were significantly associated with sedentary time and physical activity levels. Interestingly, only the activity avoidance was higher in the medium self-efficacy group compared with the low self-efficacy group, whereas the high self-efficacy group achieved better results for both activity and pain avoidance than the low self-efficacy group. These findings support the relevance of activity avoidance, which is reported in the study by Pastor-Mira et al. [24], who found that, for low physical activity, the activity avoidance behavior was a mediator of self-efficacy to explain physical activity levels. Furthermore, previous studies have reported that activity avoidance patterns are associated with worse daily functioning [23,38].

The previous study by Pastor-Mira et al. [24] only evaluated the role of activity avoidance, while the current study has also analyzed the role of persistence and pacing. These variables are relevant to explain the activity/inactivity of people. The pacing behavior is based on strategies, such as slowing down, breaking tasks into smaller and manageable tasks, or taking rests in order to increase the levels of activity, preserve energy for activities, or simply to reduce pain levels [28,39]. The current study found that light physical activity was significantly and inversely related to the pacing for pain reduction and not for increasing activity or conserving energy. That means that those with lower scores in pacing for pain reduction achieved higher levels of light physical activity. As expected, the pain-related strategy is crucial in women with fibromyalgia. This is in line with the complex concept of pacing that has been previously suggested in the scientific literature [28,38]. In this regard, pacing could be considered a strategy to increase activity levels and can be related to better psychological functioning, but it is also associated with higher pain and disability levels [38].

On the other hand, the concept of persistence is linked to the continuation of activities until it is completed, despite the aversive stimulus (pain in the case of fibromyalgia) [40,41]. Results showed that excessive persistence was related to light physical activity levels, while task-contingent persistence was related to sedentary levels (inverse relation) and directly to physical activity levels (light and moderate). However, pain-contingent persistence was not significantly associated with any of the types of physical activity. In the case of persistence, the group with low self-efficacy for physical activity had the poorest scores. This is in line with the results collected in the review by Andrews et al. [38], showing that task persistence is related to lower levels of pain and higher levels of both physical and psychological functioning.

Two limitations of this study should be mentioned. First, data from accelerometers had some failures in the devices and troubles with the signals, which reduced the total sample of the study and limited the possibilities of other analyses. The second limitation is related to the absence of men in the sample, which is mainly due to the higher proportion of diagnoses among women and the potential differences between gender. Thus, the results should not be generalized to all fibromyalgia patients.

Despite these limitations, the current study found novel links among objective physical activity, self-efficacy, and behaviors, such as avoidance, persistence, or pacing. Furthermore, researchers and professionals must consider the role of avoidance and persistence in the performance of physical activity and how those patients with low levels of self-efficacy also have low levels of task-contingent persistence and activity avoidance, while those with medium and high self-efficacy have similar levels of persistence and avoidance.

## 5. Conclusions

Self-efficacy is a key variable to achieve lower levels of sedentarism and higher levels of physical activity. Self-efficacy for walking and light physical activity is more relevant than self-efficacy for moderate or vigorous physical activity, since there were no differences between the high self-efficacy group (high levels of self-efficacy for any type of physical activity) and the medium self-efficacy group (high levels of self-efficacy for walking and light physical activity, and low levels for moderate and vigorous physical activity). Furthermore, persistence and avoidance also seem to be crucial to understand the physical activity levels of women with fibromyalgia, but further research is needed.

## Figures and Tables

**Table 1:** Main characteristics of the sample.

	Mean	SD	Median	IQR
Age (years)	56.16	9.07	57	12
BMI (m/kg^2^)	27.79	6.78	25.15	6.92
Years with pain disturbance	27.09	13.45	28	23
Years with fatigue disturbance	22.44	14.50	19	24
FIQR score	68.70	18.73	70.33	26.58

BMI: body mass index; SD: standard deviation; IQR: interquartile range; FIQR: revised fibromyalgia impact questionnaire.

**Table 2:** Correlations between physical activity and fibromyalgia variables.

	Years since Fibromyalgia Diagnosis		FIQR Overall Impact		FIQR Symptoms		FIQR Function		FIQR Total Score	
	r	*p*-Value	r	*p*-Value	r	*p*-Value	r	*p*-Value	r	*p*-Value
Sedentary time	0.234 **	0.009	0.271 **	0.002	0.167	0.064	0.310 **	<0.001	0.266 **	0.003
Light PA	−0.145	0.107	−0.211 **	0.019	−0.103	0.257	−0.200 *	0.027	−0.182 *	0.044
Moderate PA	−0.288 **	0.001	−0.303 **	0.001	−0.236 **	0.009	−0.381 **	<0.001	−0.331 **	<0.001
Vigorous PA	−0.138	0.123	−0.200 *	0.027	−0.165	0.069	−0.229 *	0.011	−0.224 *	0.013

FIQR: revised version of the Fibromyalgia Impact Questionnaire; PA: physical activity. * *p* < 0.05. ** *p* < 0.01.

**Table 3:** Correlations between physical activity levels and self-efficacy and activity patterns.

	Sedentary Time		Light PA		Moderate PA		Vigorous PA	
	r	*p*-Value	r	*p*-Value	r	*p*-Value	r	*p*-Value
Self-efficacy walking	−0.384 **	<0.001	0.328 **	<0.001	0.371 **	<0.001	0.251 **	0.005
Self-efficacy light PA	−0.344 **	<0.001	0.299 **	0.001	0.336 **	<0.001	0.204 *	0.024
Self-efficacy moderate PA	−0.294 **	0.001	0.240 **	0.008	0.292 **	0.001	0.242 **	0.008
Self-efficacy vigorous PA	−0.276 **	0.002	0.210 *	0.020	0.317 **	<0.001	0.223 *	0.014
Self-efficacy walking 30 min	−0.334 **	<0.001	0.294 **	0.001	0.354 **	<0.001	0.254 **	0.005
Self-efficacy walking 60 min	−0.259 **	0.004	0.209 *	0.021	0.302 **	0.001	0.220 *	0.015
Self-efficacy walking 90 min	−0.194 *	0.032	0.137	0.132	0.246 **	0.006	0.177	0.052
Pain avoidance	0.283 **	0.002	−0.249 **	0.007	−0.291 **	0.001	−0.159	0.085
Activity avoidance	0.257 **	0.005	−0.223 *	0.015	−0.266 **	0.003	−0.188 *	0.041
Mean avoidance	0.290 **	0.001	−0.248 **	0.006	−0.304 **	0.001	−0.203 *	0.026
Task-contingent persistence	−0.316 **	<0.001	0.332 **	<0.001	0.255 **	0.005	0.134	0.145
Excessive persistence	−0.167	0.069	0.207 *	0.023	0.088	0.337	0.037	0.686
Pain-contingent persistence	−0.141	0.124	0.144	0.118	0.072	0.432	0.041	0.654
Mean Persistence	−0.276 **	0.002	0.299 **	0.001	0.190*	0.036	0.071	0.436
Pacing increase activity	0.055	0.553	−0.055	0.552	−0.051	0.583	−0.090	0.328
Pacing conserving energy	0.108	0.236	−0.098	0.284	−0.122	0.181	−0.103	0.259
Pacing pain reduction	0.196 *	0.032	−0.244 **	0.007	−0.139	0.130	−0.094	0.306
Mean Pacing	0.127	0.162	−0.139	0.127	−0.117	0.199	−0.100	0.273

PA: physical activity. Physical activity in daily minutes. * *p* < 0.05. ** *p* < 0.01.

**Table 4:** Self-efficacy values for the three groups from the cluster analysis.

	High SE		Medium SE		Low SE	
	Mean (SD)	Median (IQR)	Mean (SD)	Median (IQR)	Mean (SD	Median (IQR)
SE walking	6.83 (1.65)	7.00 (3.00)	6.59 (1.62)	6.20 (2.50)	2.24 (1.40)	2.20 (2.10)
SE light PA	6.65 (1.46)	7.20 (2.40)	6.15 (1.47)	6.00 (2.70)	2.02 (1.39)	2.20 (2.20)
SE moderate PA	6.84 (1.42)	7.00 (2.40)	4.46 (2.11)	4.40 (2.30)	1.43 (1.44)	1.00 (2.60)
SE vigorous PA	5.81 (1.58)	5.40 (2.00)	1.55 (1.60)	1.00 (2.80)	0.37 (0.75)	0.00 (0.40)

PA: physical activity; SD: standard deviation; IQR: interquartile range; SE: self-efficacy.

**Table 5:** Results of the analyzed variables regarding the self-efficacy for physical activity levels.

	Total	High SE	Medium SE	Low SE	Kruskal Wallis	Pairwise Comparisons (*p*-Value with Bonferroni Adjustment)		
	Median (IQR)	Median (IQR)	Median (IQR)	Median (IQR)	*p*-Value	High vs. Medium SE	High vs. Low SE	Medium vs. Low SE
Sedentary time	9324 (437)	9199 (416)	9196 (533)	9434 (367)	0.006	1.000	0.045	0.024
Light PA	519 (230)	519 (199)	556 (349)	468 (239)	0.031	1.000	0.245	0.049
Moderate PA	238 (240)	281 (239)	278 (217)	207 (145)	0.004	1.000	0.023	0.022
Vigorous PA	4.75 (13)	11.75 (40)	4.75 (18)	3.75 (9)	0.016	0.246	0.016	0.440
Pain avoidance	7 (3)	6 (4)	7 (4)	7 (3)	0.035	0.158	0.029	1.000
Activity avoidance	7 (3)	5 (3)	7 (2)	8 (4)	<0.001	0.329	0.001	0.022
Mean avoidance	7 (3)	5.5 (3.5)	7 (2.5)	7.5 (2.5)	0.002	0.149	0.002	0.147
Task-contingent persistence	6 (4)	8 (3)	7 (3)	5 (4)	0.002	1.000	0.053	0.003
Excessive persistence	7 (3)	4 (5)	7 (3)	7 (3)	0.850	N.A	N.A	N.A
Pain-contingent persistence	10 (3)	10 (6)	10 (3)	10 (3)	0.312	N.A	N.A	N.A
Mean Persistence	7.7 (3)	6.33 (3.7)	8 (2.7)	7.3 (2.3)	0.028	1.000	0.133	0.049
Pacing increase activity	8 (3.50)	6 (6)	8 (4)	8 (3)	0.379	N.A	N.A	N.A
Pain conserving energy	8 (4)	7 (5)	8 (5)	7 (3)	0.511	N.A	N.A	N.A
Pacing pain reduction	8 (3)	6 (3)	8 (4)	8 (3)	0.110	N.A	N.A	N.A
Mean Pacing	8 (3.2)	6.7 (3.3)	8.3 (3.3)	8 (2.7)	0.356	N.A	N.A	N.A
FIQR overall impact	1 (8)	11 (16)	11 (9)	17 (4)	<0.001	1.000	0.003	<0.001
FIQR symptoms	36 (11.5)	29 (22.5)	33.5 (10.5)	38.5 (11)	0.006	0.614	0.014	0.079
FIQR function	21 (8.5)	15.7 (7.3)	19.3 (9.3)	22.7 (4.7)	<0.001	0.203	<0.001	0.004
FIQR total score	70.2 (24.5)	50.3 (47.7)	61.7 (25.2)	76.3 (20.2)	<0.001	0.675	0.001	0.002

SD: standard deviation; IQR: interquartile range; FIQR: revised version of the Fibromyalgia Impact Questionnaire; SE: self-efficacy.

## Data Availability

MDPI Research Data Policies at https://www.mdpi.com/ethics accessed on 13 September 2022.
